# Implication of Apoptosis for the Pathogenesis of *Trypanosoma cruzi* Infection

**DOI:** 10.3389/fimmu.2017.00518

**Published:** 2017-05-09

**Authors:** Débora Decote-Ricardo, Marise P. Nunes, Alexandre Morrot, Celio G. Freire-de-Lima

**Affiliations:** ^1^Instituto de Veterinária, Universidade Federal Rural do Rio de Janeiro, Seropédica, Brazil; ^2^Instituto Oswaldo Cruz, Fundação Oswaldo Cruz, Rio de Janeiro, Brazil; ^3^Instituto de Microbiologia Paulo de Góes, Universidade Federal do Rio de Janeiro, Rio de Janeiro, Brazil; ^4^Instituto de Biofísica Carlos Chagas Filho, Universidade Federal do Rio de Janeiro, Rio de Janeiro, Brazil

**Keywords:** *Trypanosoma cruzi*, apoptosis, immunomodulation, infection, macrophages

## Abstract

Apoptosis is induced during the course of immune response to different infectious agents, and the ultimate fate is the recognition and uptake of apoptotic bodies by neighboring cells or by professional phagocytes. Apoptotic cells expose specific ligands to a set of conserved receptors expressed on macrophage cellular surface, which are the main cells involved in the clearance of the dying cells. These scavenger receptors, besides triggering the production of anti-inflammatory factors, also block the production of inflammatory mediators by phagocytes. Experimental infection of mice with the parasite *Trypanosoma cruzi* shows many pathological changes that parallels the evolution of human infection. Leukocytes undergoing intense apoptotic death are observed during the immune response to *T. cruzi* in the mouse model of the disease. *T. cruzi* replicate intensely and secrete molecules with immunomodulatory activities that interfere with T cell-mediated immune responses and secretion of pro-inflammatory cytokine secretion. This mechanism of immune evasion allows the infection to be established in the vertebrate host. Under inflammatory conditions, efferocytosis of apoptotic bodies generates an immune-regulatory phenotype in phagocytes, which is conducive to intracellular pathogen replication. However, the relevance of cellular apoptosis in the pathology of Chagas’ disease requires further studies. Here, we review the evidence of leukocyte apoptosis in *T. cruzi* infection and its immunomodulatory mechanism for disease progression.

## Introduction

Chagas disease or American trypanosomiasis was discovered by Carlos Chagas in the early twentieth century. The disease is caused by the protozoan parasite *Trypanosoma cruzi*. It is estimated that 18–20 million people in Latin America are infected with *T. cruzi*, and approximately 100 million people are living in areas at risk of infection (1). *T. cruzi* is an obligate intracellular parasite that infects a variety of the mammalian host cells but shows preference for cells of the macrophage and muscle lineage. The biological cycle in man has two evolutionary forms (1) trypomastigotes, the flagellate form of the parasite that invades cells, where they differentiate into (2) amastigotes that have no free flagellum and replicate inside almost all nucleated cells of the vertebrate host (2). Soon after infection, trypomastigotes have the ability to escape the parasitophorous vacuole and differentiate into amastigotes in the host cell cytoplasm. After several rounds of binary divisions inside the infected cells, amastigotes differentiate to trypomastigotes forms, lyse the infected cells, and reinvade adjacent cells (3, 4).

An important successful factor that allows *T. cruzi* to survive in the vertebrate host is the evasion of the cellular immune response (5–7). Many studies in this area describe the existence of different strategies developed by the parasite to modulate the immune response of the vertebrate host in its favor (4, 8–12).

One of the most efficient mechanisms that *T. cruzi* parasites use to establish a persistent infection is the induction of T and B cell apoptosis, and such process has immunomodulatory effects on the host immune response (8, 13).

## Apoptosis as a Hallmark of *T. cruzi* Infection

Apoptosis is crucial for normal tissue homeostasis and for modulation of immune response. During parasitic infections, apoptosis or programmed cell death can be triggered by antigens, factors secreted and/or released by pathogens, by chronic infection, and by intense cellular activation (14–16). Recent evidences have shown that in order to survive in their hosts, intracellular protozoan parasites have to limit the defense mechanisms and are taking advantage of cell death to facilitate parasite spreading. It was described that *T. cruzi* infection triggers activation-induced cell death (AICD) of CD4^+^ T lymphocytes during the acute phase of infection (8, 17). The AICD occurs after stimulation with anti-TCR and anti-CD3 agonist antibodies *in vitro*. When cultured in the presence of anti-Fas agonist antibody, CD4^+^ T cells from infected mice, but not from normal mice, undergo apoptosis (17, 18).

The interaction between the Fas molecule (CD95) and its ligand, the Fas ligand (FasL) molecule (CD95L) induces death by apoptosis (19). The involvement of Fas/FasL molecules was confirmed during the acute phase of *T. cruzi* experimentally infected mice. It was found that there is an increased expression and function of the Fas/FasL in CD4^+^ T cells, and cells from FasL-deficient mice (*gld* mice) do not undergo AICD during *T. cruzi* infection (20). It has been suggested that AICD could have a deleterious role in *T. cruzi* infection, causing early elimination of effector T cells and supporting parasite escape (13). In agreement with these results, Rodrigues et al. (21) observed high levels of FasL in the serum of *T. cruzi* chronically infected patients (21). In addition, studies by Guillermo et al. (22) and Vasconcelos et al. (23) have revealed more information about the kinetics of Fas/FasL expression and T lymphocytes apoptosis during *T. cruzi* experimental infection (22). Splenic CD4^+^ and CD8^+^ T cells showed an upregulation in CD95/CD95L expression in a time-dependent manner during *T. cruzi* acute infection, which was associated with activation-induced cell death (AICD). *In vivo* injection of anti-FasL, but not anti-TNF-alpha or anti-TRAIL antibodies, blocked activation-induced cell death of CD8^+^ T cells, improved type 1 immune responses, and reduced the infection severity as estimated by parasitemia (22, 23). Recently, Chaves et al. (24) demonstrated that T cell apoptosis was related to decreased cell proliferative and modulation of genes associated with apoptosis and caspase family receptors in chagasic patients with heart problems. Thus, the authors concluded that the T cell death was interfering with the clinical manifestations of the disease (24).

Infection caused by *T. cruzi* results in polyclonal lymphocyte activation (24–27), which, by itself, triggers T cell apoptosis (28, 29). *T. cruzi* released molecules such as *trans*-sialidase, an enzyme that catalyzes the transfer of exogenous sialic acid residues from the host acceptor molecules on the *T. cruzi* surface, induces a strong cell death by apoptosis and resulted in increasing parasite infectivity (30–32).

The *T. cruzi* surface expresses an unusual family of glycoinositolphospholipid (GIPL) molecules that are present in all *T. cruzi* evolutionary forms (33). Studies from our group have shown that in the presence of the cytokine IFN-γ, the ceramide portion of the GIPL induced intense macrophage apoptosis, independent of nitric oxide production. This effect was not observed when macrophages were treated with intact *T. cruzi* GIPL or with the GIPL-derived glycan chain. In *T. cruzi*-infected macrophages, apoptosis also increased the release of infective trypomastigotes and spheromastigotes (34). The pro-apoptotic action of ceramide had been described in previous studies, in which permeable ceramides were shown to promote a strong cytotoxic effect of this molecule (35, 36). Recently, the α-galactosylceramide molecule derived from *T. cruzi* induced NK T cell anergy and IL-33-mediated myeloid-derived suppressor cell accumulation (37).

Clearance of apoptotic cells involves the recognition of the dying cell by the phagocytes, internalization, the immunological consequences to host–parasite interaction, and pathogenesis of disease (38). In recent years, the term “efferocytosis” has been introduced to specifically refer to the engulfment or phagocytosis of apoptotic cells (39–42). Macrophages undergo specific molecular and functional changes upon encounter, interaction with, and uptake of apoptotic cells (efferocytosis) that control both phagocytosis and immune signaling (42).

*In vitro* studies showed that engulfment of apoptotic CD4^+^ T lymphocytes from infected mice by *T. cruzi* experimentally infected macrophages exacerbate parasite replication. When apoptosis of CD4^+^ T cells from infected mice was blocked with anti-FasL mAb, parasite growth was also blocked. Prevention of parasite growth was observed in a transwell coculture system, where CD4^+^ T cells were cultured and separated from infected macrophages, showing that the contact and phagocytosis of apoptotic cells ensures parasite replication. This finding was also associated with decreased IFN-γ production, suggesting that AICD was occurring on Th1 CD4^+^ cell population (18). Apoptotic cells trigger production of anti-inflammatory cytokines such as IL-10 and TGF-β by the phagocytes (41, 43–47). The process of apoptotic cells uptake by *T. cruzi*-infected macrophages is associated with the release of TGF-β IL-10 and prostaglandin PGE_2_ (8, 9, 12). Together, these mediators would deactivate macrophages and favor the growth of *T. cruzi* amastigotes and other intracellular parasites (8, 18, 48).

Phagocytic cells express on the cell surface a group of receptors that actively participate in the recognition and capture of apoptotic bodies, the vast majority of these receptors have affinity to phosphatidylserine (PtdSer) expressed by apoptotic cells (49). The integrin αVβ3 is one of these receptors, which recognizes PtdSer through molecules that act as bridges, such as lactadherin, glycoprotein produced by phagocytes, and the fat globule of opsonin-factor-8 EGF (MFG-E8) (50). In addition, the αvβ3 integrin express on phagocytes can bind to thrombospondin, helping the recognition of PS on the surface of apoptotic cells (51). Other apoptotic cell recognition receptors, such as the family of receptor tyrosine kinases Tyro3, Axl, and MerTK (TAM), also need to bind to bridge molecule to interact with PtdSer on the surface of apoptotic cells (52–54).

Because *T. cruzi* is an intracellular pathogen that can not produce putrescine, and is an auxotrophic parasite for polyamines, because of its incapacity to produce putrescine due to the lack of both, ornithine decarboxylase (ODC) and arginine. *T. cruzi* needs to capture exogenous putrescine for the proliferation of amastigote forms within the cells (55, 56). Besides the fact that binding of apoptotic lymphocytes to α_V_β_3_ expressed by macrophages results in secretion of PGE_2_ and TGF-β, the engulfment of apoptotic cells is also followed by induction of ODC and synthesis of putrescine: this functions as a growth factor for intracellular forms of *T. cruzi* (8, 9). In addition, this deleterious effect of apoptotic cells is eradicated by inhibitors of prostaglandin synthesis (NSAIDs drugs) and neutralizing antibodies for TGF-β (11, 12). Injection of apoptotic cells increases parasitemia *in vivo*, and treatment with the cyclooxygenase inhibitors aspirin or indomethacin reduces parasitemia (8).

The use of NSAIDs in *T. cruzi* experimental infection has been used by different groups. However, it is important to emphasize that the use of cyclooxygenase blockers in experimental infection may lead to conflicting results, depending on the experimental model (8, 11, 57, 58). Recently, it was described that aspirin in low doses decreased mortality, parasitemia, and heart damage in *T. cruzi*-infected mice, and they suggested that the protective effect was established to the generation of anti-inflammatory mediator 15-epi-LXA4 (59). However, when aspirin was given in high doses, this protective effect disappeared (59). Also, it has been characterized that the use of COX inhibitors inhibits *T. cruzi* infection of murine cardiac cells (60). The results presented by Michelin and collaborators suggest that the prostaglandins produced mainly by the activation of the COX-2 enzyme favor the immunosuppression of the acute phase of infection (61). These data reinforce our hypothesis that NSAIDs may somehow favor the immune response of the vertebrate host (8, 11).

The contribution of apoptotic T cell pathways in the outcome of *T. cruzi* infection *in vivo* was addressed by injection of caspase 8 inhibitor or by the use of caspase 8-deficient mice. However, blockade of the initiator caspase 8 *in vivo* was unable to inhibit apoptosis, and mice that have received treatment demonstrated a profound CD8^+^ T cell depletion. Furthermore, caspase 8-deficient mice upregulated Th2 cytokine responses and increased susceptibility to *T. cruzi* infection (62). Therapy with zIETG (caspase 8 inhibitor), initiated 4 days after infection, resulted in inhibition of T cell expansion and increased parasitemia (62). In contrast, administration of zVAD, a pan caspase inhibitor (or anti-FasL antibody) initiated at day 7 after *T. cruzi* infection was shown to reduce T cell death, promotes type 1 immune response and reduced parasitemia. Spleen cells produced more IFN-γ when stimulated with parasite antigens, while peritoneal macrophages showed a reduced parasite load (63). The number of inflammatory cells in the hearts of acutely infected mice injected with zVAD was not affected (63). Treatment with anti-FasL mAb starting at 11 days after infection, but not anti-TNF or anti-TRAIL antibodies, protect CD8 T cells from AICD, improved cytokine production by CD4 T cells, activate CD8 T cells, upregulate Fas expression by CD8 T cells earlier than CD4 T cells, and decreased parasitemia (22). Mice vaccinated with an adenoviral vector expressing two *T. cruzi*-dominant epitopes improved CD8 T cell functionality and decreased parasitemia after parasite challenge, a phenotype attributed to the lack of CD95 expression in parasite-specific CD8 T cells (23). Recently, Cabral-Piccin and collaborators demonstrated that treatment of *T. cruzi*-infected mice with anti-FasL prevents CD8^+^ T lymphocytes apoptosis, upregulates type 1 responses to parasite antigens, and reduces macrophages infection when cocultured with CD8 T cells (64). Further analysis showed that injection of anti-FasL mAb resulted in a polarized M1 macrophage phenotype, both *in vitro* and *in vivo*. The authors suggested that rescuing CD8 T cells from death with anti-FasL treatment prevents the negative effects of efferocytosis on macrophage activation.

## Concluding Remarks

The role of T cells-mediated immune response in controlling intracellular protozoan parasite infection is well established. In the acute phase of infection, *T. cruzi* proliferates within the cells of the vertebrate host, besides producing and secreting molecules with immunomodulatory activities that obstruct the immune response mediated by T lymphocytes. The final result of this important modulatory mechanism is the propagation of *T. cruzi* to different organs of the vertebrate host (13). It is also clear that T cell apoptosis occurs during human and experimental infection with *T. cruzi* (8, 24). Some studies showed evidences that parasites have adapted to their hosts modulating or even taking advantage of cell death in order to facilitate their own survival in a hostile environment and promoting disease. In this context, elucidation of apoptotic cell-mediated signaling mechanisms would help to discover new effective therapies and vaccines.

The modulation of host immune response is an important approach in the efficacy of treatment against *T. cruzi* in the experimental acute models of Chagas disease. The quality of response could be an important factor, not only in T cell disease progression but also in chemotherapy responsiveness. Apoptosis modulation has a beneficial therapeutic effect in various cardiovascular diseases, as well as infectious diseases (65). There appears to be an association between apoptosis and heart failure, as well as in disease severity in Chagas patients, such that pharmacological use of apoptosis inhibitors could be an attractive choice for adjuvant therapy in the chronic treatment phase; this restores an efficient T cell immune response to parasite infection, thus diminishing the pathological signals of disease.

## Author Contributions

DD-R, MN, AM, and CGF-de-L wrote the paper. All authors read and approved the final version of the manuscript.

## Conflict of Interest Statement

The authors of the review declare that the study was conducted in the absence of financial or commercial relationships that could be construed as a potential conflict of interest.
